# The British Journals: Surgery

**Published:** 1840-01

**Authors:** 


### SURGERY.

### *Case of Popliteal Aneurism, successfully treated by Pressure.* By E. G. Brunker, M.D., Surgeon to the County of Louth Infirmary.

Owen Matthews, aged thirty-two years, was admitted into the Louth Infirmary, on the 20th August, 1839. His occupation for some years past had been very laborious, having been employed on board a ballast vessel in the Thames. His attention was first accidentally attracted to a pulsating tumour in the left ham, fifteen months ago, but he cannot recollect any particular sensation at its first appearance. He continued to labour as usual for some time after. On admission, he had all the appearance of rude health, and on close examination, no disease of any internal organ could be discovered. Respiration natural—pulse 72, regular—cordiac sounds healthy—has not had palpitation.

On examining the ham, the entire space was observed to be completely filled up, by a pulsating tumour, beating synchronously with the heart—soft—compressible—but becoming distended on the removal of the pressure. The pulsation was very perceptible in the whole extent of the tumour. No *bruit de soufflet*, or other morbid sound was discoverable. Pressure being made on the femoral artery in the groin, the pulsation in the tumour instantly ceased, and as immediately returned on its removal. During the pressure, the size of the tumour was sensibly diminished, but resumed its former dimensions when the circulation was restored. No pulsation discoverable in either anterior or posterior tibial arteries. The patient did not experience any pain in the tumour, but merely an uneasy sensation in some of the toes and foot, which were occasionally cold and numb. He was ordered to remain in bed, and was placed on low diet.

Two days after admission, a piece of dry sponge was placed over the aneurism, and retained in situ by a roller rather loosely applied.

On Tuesday, 27th August, the infirmary was visited by Dr. Foss, of the 38th regiment, to whom I showed the case. On removing the bandage and sponge, I was greatly surprised to find that the tumour was much diminished in size, and that no pulsation whatever could be discovered, either by the hand or the stethoscope. The tumour itself was much firmer, and did not fill up the popliteal space, as it did when last examined, two days previously, by me as well as by my friend, Dr. Browne, of this town. The pressure was ordered to be continued, and the utmost quietness in the recumbent posture observed. The patient, a few days after this, left the hospital, contrary to my wish and advice.

September 12th. I this day saw Matthews, who says he has kept himself very quiet since he left the hospital, and has continued the use of the sponge and bandage. On removing them, I could scarcely discover any traces of the former tumour in the ham, and no pulsation whatever was to be felt. There is a very obscure pulsation in the anterior tibial artery, which could not before be discovered on very careful search. The uneasy sensations about the foot, give him now very little annoyance, and he walks without pain or lameness.

I have no doubt in my own mind, that the moderate pressure occasioned by the sponge and bandage, have caused the total obliteration of the sac of the aneurism, and thus this patient has been saved the risk inseparable from the application of a ligature to the femoral artery, which operation I should have had recourse to, had not the more simple means I thought it but fair to make trial of in the first instance been attended with the success I have mentioned.

*Dublin Medical Press. Oct.* 16, 1839.

